# Apoptosis Triggered by ORF3 Proteins of the *Circoviridae* Family

**DOI:** 10.3389/fcimb.2020.609071

**Published:** 2021-02-02

**Authors:** Yanting Zhang, Xingcui Zhang, Anchun Cheng, Mingshu Wang, Zhongqiong Yin, Juan Huang, Renyong Jia

**Affiliations:** ^1^ Research Center of Avian Disease, College of Veterinary Medicine, Sichuan Agricultural University, Chengdu, China; ^2^ Institute of Preventive Veterinary Medicine, College of Veterinary Medicine, Sichuan Agricultural University, Chengdu, China; ^3^ Key Laboratory of Animal Disease and Human Health of Sichuan Province, College of Veterinary Medicine, Sichuan Agricultural University, Chengdu, China

**Keywords:** *Circoviridae*, open reading frame 3 protein, apoptosis, signaling pathways, interaction proteins

## Abstract

Apoptosis, a form of the programmed cell death, is an indispensable defense mechanism regulating cellular homeostasis and is triggered by multiple stimuli. Because of the regulation of apoptosis in cellular homeostasis, viral proteins with apoptotic activity are particular foci of on antitumor therapy. One representative viral protein is the open reading frame 3 (ORF3) protein, also named as apoptin in the *Circoviridae* chicken anemia virus (CAV), and has the ability to induce tumor-specific apoptosis. Proteins encoded by ORF3 in other circovirus species, such as porcine circovirus (PCV) and duck circovirus (DuCV), have also been reported to induce apoptosis, with subtle differences in apoptotic activity based on cell types. This article is aimed at reviewing the latest research advancements in understanding ORF3 protein-mediated apoptosis mechanisms of *Circoviridae* from three perspectives: subcellular localization, interactions with host proteins, and participation in multiple apoptotic signaling pathways, providing a scientific basis for circovirus pathogenesis and a reference on its potential anticancer function.

## Introduction

The *Circoviridae* family, whose members are considered to cause fatal diseases in birds and pigs, is composed of two recognized genera: *Circovirus* and *Cyclovirus (* Rosario et al., 2017). Chicken anemia virus (CAV), the only bird virus member of the C*yclovirus* family, leads to atrophy of bone marrow hematopoietic tissue and lymphatic tissues in young chickens (Taniguchi et al., 1983). The *Circovirus* genus contains pathogenic viruses of vertebrates, such as beak and feather disease virus (BFDV), goose circovirus (GoCV), duck circovirus (DuCV) (Hattermann et al., 2003), and porcine circovirus (PCV) (Ha et al., 2020). The presence of *Circovirus* in invertebrates has also been reported (Wang et al., 2018). Infections with any of these viruses can potentially cause fatal diseases (Todd, 2000; Soike et al., 2004; Todd, 2004; Raidal et al., 2015; Palinski et al., 2016; Dennis et al., 2018; Klaumann et al., 2018; Fatoba and Adeleke, 2019), which are characterized by damage to lymphoid tissues and immunosuppression (Todd, 2000).

**Figure 1:**
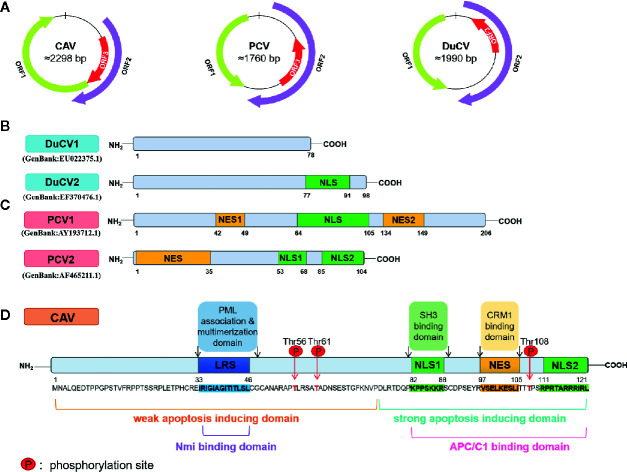
Schematic diagram of the CAV, PCV and DuCV gene structures. **(A)** The relative localizations of three major ORF proteins in CAV, PCV, and DuCV differ from each other. In particular, the ORF3 proteins of PCV and DuCV are oriented in the direction opposite that of Rep gene, in contrast to the ORF3 protein in CAV. **(B)** The ORF3 protein structures between DuCV1 and DuCV2 differ. The DuCV2 ORF3 (99 aa) is 20 aa longer than that of DuCV 1, in which an NLS has been identified. **(C)** The ORF3 structures between PCV1 and PCV2 differ. The length of the ORF3 is 315 bp in PCV2, while in PCV1, it is approximately 612 bp. The NES has been identified in the N-terminus of PCV2 ORF3 protein at residues 1–35, and a bipartite NLS was found at residues 53–68 and 85–104. **(D)** The primary structures of the CAV ORF3 protein (Los et al., 2009). The following main domains are shown in color: leucine-rich region (*blue*), phosphorylation site (*red*), bipartite nuclear localization sequences (*green*), and nuclear export sequence (*yellow*).

The members of the *Circoviridae* family are small, nonenveloped viruses with circular single-stranded DNA genomes. The length of genome is approximately 2000 bp, and it contains two major open-reading frames (ORFs)—ORF1 and ORF2, encoding Cap and Rep proteins, respectively. Cap is the sole structural protein of the virus and has a highly conserved basic amino acid sequence, indicating that Cap contains the major antigenic determinant (Nawagitgul et al., 2000; Hattermann et al., 2003). Rep is mainly associated with rolling circle replication (RCR) (Hamel et al., 1998; Luo et al., 2018). Adjacent to two Cap and Rep, the origin of replication with a stem loop structure is located in the intergenic region (Faurez et al., 2009). Especially for PCV, a Rep’ protein can be generated by alternative transcript splicing of ORF1, and the Rep-Rep’ complex is required for promoting virus replication by RCR system (Steinfeldt et al., 2002). In addition to the Rep and Cap, ORF3 of *Circovirus* encodes a protein that can participate in cell death during viral infection. In PCV, the ORF3 protein plays an important role in the pathogenesis of the virus due to its apoptotic activity *in vitro* and *in vivo*, although it is not essential for virus replication (Liu et al., 2005; Liu et al., 2006; Lin et al., 2011). In other kinds of *Circoviruses*, such as DuCV, the ORF3 protein is thought to induce apoptosis as well (Xiang et al., 2012). In *Cyclovirus*, reports have implicated that ORF3 encodes a nonstructural protein and participates in the induction of apoptosis and viral cytotoxicity in host cells (Kucharski et al., 2016) (**Figure 1A**).

Apoptosis causes a non-lytic and typically immunologically silent form of cell death (Jorgensen et al., 2017). The extrinsic pathway and intrinsic pathway in host cells, also known as the death receptor pathway and mitochondrial pathway, respectively, are regarded as the two classic apoptotic ways to initiate cell death, as both culminate in the activation of the effector caspases 3 (Elmore, 2007; Jorgensen et al., 2017). Activation of the intrinsic pathway causes mitochondrial outer-membrane permeabilization (MOMP) (Green and Kroemer, 2004; Kroemer et al., 2007) and the release of mitochondrial contents to the cytoplasm (Dewson and Kluck, 2009; Zheng et al., 2016), where cytochrome C forms an apoptosome (Cain et al., 2002; Jiang and Wang, 2004; Taylor et al., 2008; Jorgensen et al., 2017). In the extrinsic pathway, apoptosis is triggered by the binding of a specific ligand and its cognate death receptor, featured with the activation of caspase 8 and caspase 3, which ultimately causes cellular DNA cleavage (Tummers and Green, 2017). Moreover, an endoplasmic reticulum (ER) pathway can elicit cell apoptosis (Kaufman, 2002) by regulating the concentration of Ca^2+^ and activating the inositol-requiring enzyme 1 (IRE1), protein kinase-like endoplasmic reticulum kinase (PERK), and activating transcription factor (ATF) pathways, which are connected to the mitochondrial pathway (Verma and Datta, 2012). Upon activation of the different intermediate molecules in a signaling cascade, each of these pathways comes across at the same terminal caspase activation step and generally leads to cleavage of various proteins (Ghobrial et al., 2005). The viral ORF3 protein is considered to possess potential apoptotic activity because it interacts with proteins in these apoptosis pathways to induce cell death.

## The Structure of the ORF3 Proteins in the *Circoviridae* Family

In different viruses, such as PCV, DuCV, and CAV, ORF2 is commonly located on the sense strand, and ORF1 and ORF3 are located on the antisense direction. Specifically, for PCV and DuCV, ORF3 is oriented in the opposite direction of ORF2, which is different from CAV (**Figure 1A**). In PCV2, the ORF3 protein was first described as nonstructural (Liu et al., 2005). Moreover, the length of ORF3 is 315 bp in PCV2 and approximately 612 bp in PCV1, which means that the ORF3 protein in PCV2 is truncated (**Figure 1C**). In contrast, the CAV ORF3 protein, specifically termed as apoptin, is a compact polypeptide consisting of 121 amino acids (Leliveld et al., 2004). In addition, the amino acid identity of ORF3 in PCV1 and PCV2 is only about 61.5% (Finsterbusch and Mankertz, 2009). The results of sequence comparison analyses revealed that DuCV can be divided into two genotypes: DuCV1 and DuCV2 (Wang et al., 2011; Zhang et al., 2013; Wen et al., 2014) (**Figure 1B**), and within the same genotype, the ORF3 homology reaches 95.8%–100%. Due to a T/A difference at nucleotide 236 in DuCV1, ORF3 protein (78 aa) is truncated by a premature stop codon. In other words, similar to PCV1 and PCV2, ORF3 protein of DuCV1 is 20 aa shorter at C-terminus than that of DuCV2 (Wu et al., 2018).

Notably, the ORF3 protein of CAV has a short hydrophobic leucine-rich sequence (LRS) at the N-terminus (aa 33-46) mediating self-association and binding of promyelocytic leukemia (PML) protein (Heilman et al., 2006; Janssen et al., 2007) and multiple other cellular partners (**Figure 1D**). Upon their formation, PML nuclear bodies (PML NBs) recruit the anaphase-promoting complex/cyclosome (APC/C) to these subnuclear structures (Heilman et al., 2006), which takes part in the process of apoptosis. In addition, through the interaction of the proline-rich hydrophobic regions in the N-terminus (aa 1-69), this ORF3 protein forms globular complexes composed of 30-40 monomers (Leliveld et al., 2003a; Leliveld et al., 2003b), which may play important roles in apoptosis regulation.

## The Nuclear Localization Signals of ORF3 Proteins

Nuclear localization signals (NLSs) contribute to the understanding of protein subcellular localization. NLSs are classified as either monopartite, characterized by a cluster of basic residues, or bipartite, characterized by two clusters of basic residues separated by several other residues (Canela-Pérez et al., 2019).

A bipartite NLS in CAV ORF3 is composed of NLS1 (aa 82-88) and NLS2 (aa 111-121) (**Figure 1D**). Due to the recognition of the NLS by members of the importin (IMP) family, the ORF3 protein undergoes active nuclear import. Therefore, in both normal and transformed cells, the ORF3 protein can shuttle in and out of the cell nucleus owing to its NLS and central nuclear export signal (NES) at residues 97-105 (Wang et al., 2004; Poon et al., 2005; Heilman et al., 2006). Moreover, the NLS activity of the CAV ORF3 protein was also found to be regulated by intramolecular masking (Wagstaff and Jans, 2006). For PCV1, an LRS at residues 42-49 and a strong probability of an NES at residues 134-149 in ORF3 were predicted (Hough et al., 2015) (**Figure 1C**). Interestingly, compared with the sequence of CAV ORF3, the sequence alignment of PCV1 showed an additional NES sequence overlapping a region just upstream of the predicted area for the C-terminal NES (residues 127-136) (Hough et al., 2015). For PCV2, the NLS was confirmed to be in two halves of the C-terminal region at residues 53–68 and 85–104 (Lin et al., 2011). In the N-terminal of the ORF3, an NES motif is located at residues 1–35 (Gu et al., 2016). Furthermore, a variant monopartite type of NLS has been identified at the C-terminal 77–91 residues in the DuCV 2 ORF3, which is essential for its nuclear localization (Wu et al., 2018) (**Figure 1B**).

## The Regulation of ORF3-Induced Apoptosis

In the genome of *Circoviridae* family members, ORF3s have been recognized as encoding functional proteins connected to apoptosis (Liu et al., 2005; Liu et al., 2006; Karuppannan et al., 2009; Karuppannan and Kwang, 2011; Lin et al., 2011; Xiang et al., 2012; Kucharski et al., 2016). It appears that the subcellular localization of ORF3 proteins and their interactions with specific signaling proteins play crucial roles in selective toxicity prior to the induction of apoptosis.

### The Contribution of ORF3 Subcellular Localization to Apoptosis

The subcellular localization of ORF3 proteins is closely related to their NLSs. For instance, in a majority of tumor and transformed cells, CAV ORF3 proteins primarily accumulate in the nucleus (Danen-Van Oorschot et al., 1997; Noteborn, 2009). In addition, the nuclear accumulation of ORF3 proteins in PCV2 and DuCV2 has been confirmed (Lin et al., 2011). Nevertheless, the DuCV1 protein without an NLS is dispersed in the cytoplasm (Wu et al., 2018).

The CAV NES and NLS, residues 74–121 in the C-terminus constitute tumor cell-specific nuclear targeting signals (Danen-Van Oorschot et al., 2003; Kuusisto et al., 2008). Thus, the ORF3 protein has selective toxicity that induces apoptosis in various transformed cells by gathering in the nucleus, whereas in normal cells, the apoptotic activity of the ORF3 protein disappears because of nuclear accumulation impairment (Danen-Van Oorschot et al., 1997; Poon et al., 2005; Helck et al., 2008; Zhao et al., 2013). There are reports of CAV ORF3 protein toxicity towards SV40-transformed fibroblasts and UV-irradiated cells from individuals with hereditary cancer-prone syndromes as well (Danen-Van Oorschot et al., 1997; Zhang et al., 1999; Guelen et al., 2004). Only one report indicates it’s toxicity towards some non-cancerous cells, such as 1BR3 normal human diploid fibroblasts and the early passage secondary culture of normal human embryonal lung fibroblasts 6689, which has no further research about it’s mechanism. And most studies show that apoptosis induction by ORF3 proteins is confined to a broad panel of human tumor cells (Backendorf et al., 2008). Therefore, this study focuses mainly on the different mechanisms of CAV ORF3 protein in cancer cells and in normal cells. In addition, the nuclear localization of the PCV2 ORF3 is correlated with a triggered apoptotic response in porcine peripheral blood mononuclear cells (PBMCs) (Lin et al., 2011). Although the cytoplasmic localization of the PCV1 ORF3 protein does not differ in primary and transformed cells, the ability of ORF3 protein to induce apoptosis selectively relies on the transformation status of the cell (Hough et al., 2015). Similar to ORF3 proteins of CAV and PCV, DuCV ORF3 protein has also been proven to have apoptotic activity (Xiang et al., 2012), implying that it may play a vital role in the pathogenesis of DuCV (Wu et al., 2018). A study showed that the nuclear localization of the DuCV2 ORF3 protein enhanced its apoptotic activity, compared with that of DuCV1, whose ORF3 protein is dispersed in the cytoplasm (Wu et al., 2018). And the analysis of the apoptotic activities of ORF3 proteins in DuCV1 and DuCV2 suggested that the 20 C-terminal residues in the DuCV2 ORF3 self-inhibit the virus-induced apoptotic activity (Wu et al., 2018); however, the mechanism of this inhibition is unclear. In addition, there is a precise difference in the pathogenesis of these two genotypes.

**Figure 2:**
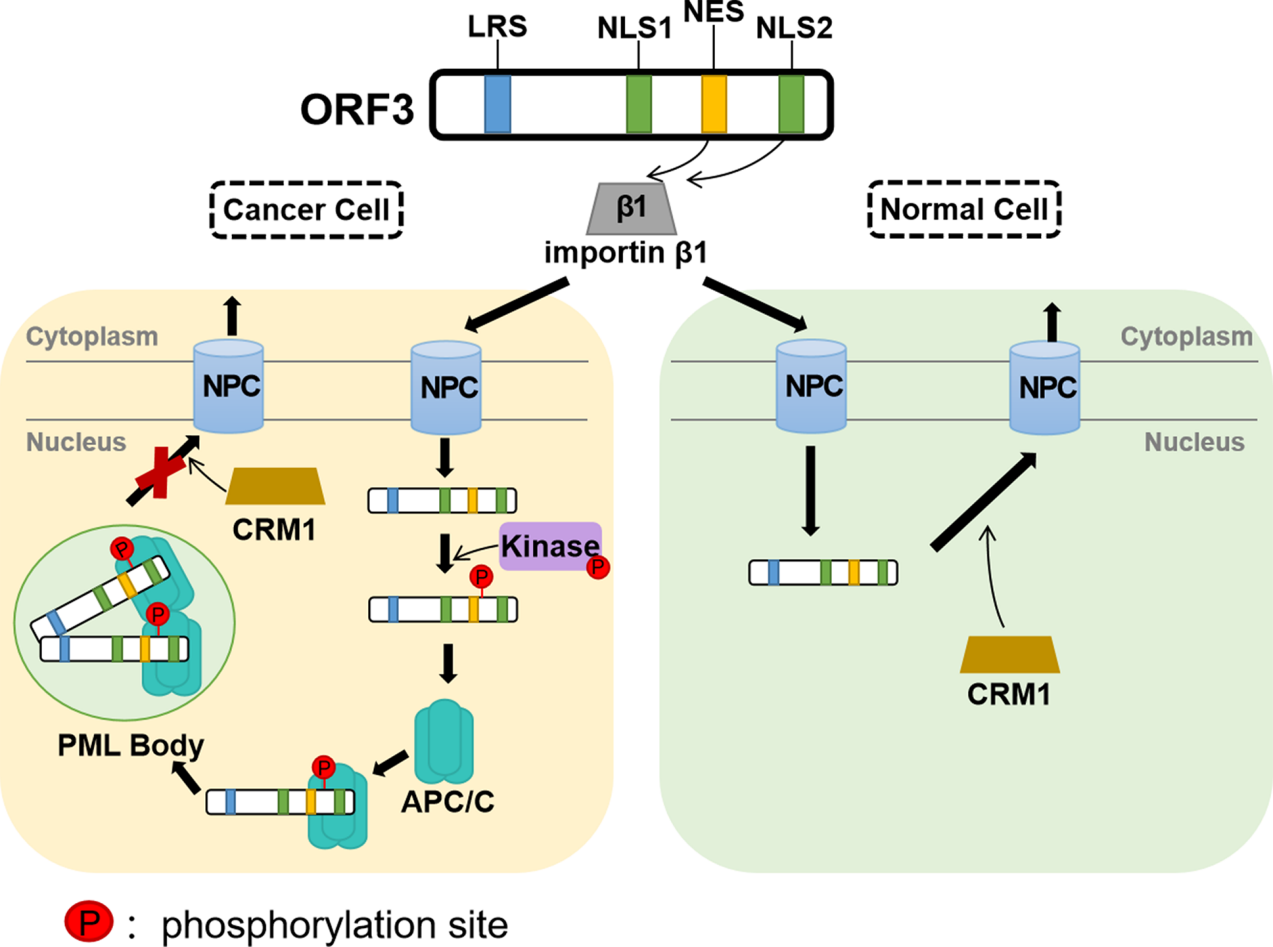
A simulation model of the CAV ORF3 protein associated with various binding partners and its different effects on signal transduction pathways. The ORF3 protein can associate with importin β1, which facilitates its translocation from the cytoplasm to the nucleus. In tumor cells, the ORF3 protein phosphorylated by Kinase, interacts with a subunit of APC/C called APC1 and directly interacts with PML to induce the formation of PML nuclear bodies. The NES of the ORF3 protein is functional in a CRM1-dependent fashion in normal but not in tumor cells, which indicates that in tumor cells, the ORF3 protein is unable to be driven out of the nucleus with the NES out of function when CRM1 inhibited. This hypothetical model may not apply to non-tumor cells in 1BR3 normal human diploid fibroblasts and the early passage secondary culture of normal human embryonal lung fibroblasts 6689 (Guelen et al., 2004).

Exploring the tumor cell-specific nuclear signal of the CAV ORF3 protein, evidence from other studies suggests that the ORF3 protein is phosphorylated robustly in a broad panel of tumor cells but negligibly in normal cells (Rohn et al., 2002). Tumor-specific phosphorylation depends on the phosphorylation site threonine 108 (Thr108), which allows its interaction with other proteins and modification by kinases. Because Thr108 phosphorylation inhibits the activity chromosome region maintenance 1 (CRM1), the ORF3 protein with the NES motif, which depends on a functional CRM1, cannot be driven out of the nucleus in tumor cells (Poon et al., 2005) (**Figure 2**). Although the adjacent threonine (Thr107) was also found to serve as a compensatory phosphorylation site in the event of Thr108 de-phosphorylation, the activity of the ORF3 protein is diminished (Rohn et al., 2005; Lanz et al., 2012).

Further investigation confirmed that the DNA damage response (DDR) regulates the nuclear localization and apoptotic effect of CAV ORF3 through checkpoint kinase1/2 (Chk1/2), which is mediated by the phosphorylation of Thr56 and Thr61 during viral replication (Kucharski et al., 2016; Feng et al., 2020). Upon induction of DNA damage, the ORF3 protein expressed in primary cells is translocated to the nucleus, but in transformed cells, the inhibition of DDR signaling results in the cytoplasmic localization of the protein (Kucharski et al., 2011). To date, the specific molecular mechanisms by which the ORF3 protein moves between subcellular localizations and mediates apoptosis remain to be determined.

### Proteins Interacting With ORF3

Since DuCV cannot be isolated and cultured *in vitro*, researchers have focused more on CAV and PCV; hence the mechanisms of DuCV, particularly the ORF3 protein, remain unclear. The identification of ORF3-associating proteins will guide us to a greater understanding of the mechanism of ORF proteins in apoptosis induction.

#### CAV

Recently, a number of cellular proteins have been found to interact with the CAV ORF3 protein, including PI3-K (Maddika et al., 2007), PKCβ1 (Jiang et al., 2010; Bullenkamp et al., 2015), CDK1 (Maddika et al., 2009; Zhao et al., 2013), Nmi (Sun et al., 2002), DEDAF (Danen-van Oorschot et al., 2004), APC/C (Teodoro et al., 2004), Hippi (Cheng et al., 2003), Ppil3 (Huo et al., 2008), FADD (Guelen et al., 2004), Bcl10 (Guelen et al., 2004), Hsp70, and Hsc (Chen et al., 2011; Yuan et al., 2013). Among these proteins, PI3-K, Nmi, Hippi, Ppil3, FADD, Bcl10, and Hsp70, were confirmed to interact with the ORF3 protein in the cytoplasm. In the nucleus, the ORF3 protein interactions with DEDAF, PML, and APC were identified (**Table 1**).

**Table 1:** Molecules that interact with CAV and PCV ORF3 proteins.

Viruses	Cellular localization	Interacting molecules	Biological effects	References
CAV	Nucleus	PML	The disruption of the interaction between the PML and ORF3 proteins does not affect their cytotoxicities	(Heilman et al., 2006)
		CDK1 and CDK2	Induce ORF3 Thr108 phosphorylation; regulate ORF3 subcellular localization	(Maddika et al., 2009; Zhao et al., 2013)
		PKC β	Make ORF3 phosphorylated	(Jiang et al., 2010; Bullenkamp et al., 2015)
		APC1	Induces cell cycle arrest in mitosis	(Teodoro et al., 2004)
		DEDAF	Increases apoptosis	(Danen-van Oorschot et al., 2004)
	Cytoplasm	Nmi	May alter the activity of Nmi	(Sun et al., 2002)
		Importin β1	Facilitates ORF3 nuclear translocation	(Poon et al., 2005; Kuusisto et al., 2008)
		PI3K	Activates PI3K and Akt; facilitates Akt nuclear translocation	(Maddika et al., 2007; Maddika et al., 2008)
		Hippi	Co-localizes with ORF3 protein in the cytoplasm of non-cancerous cells, whereas in tumor cells, the ORF3 protein migrates to the nucleus and Hippi remains in the cytoplasm	(Cheng et al., 2003)
		Ppil3	Facilitates cytoplasmic localization of ORF3	(Huo et al., 2008)
		FADD	Co-localizes in so-called death effector filaments	(Guelen et al., 2004)
		Bcl10	Co-localizes to cytoplasmic filaments; regulates apoptosis and NF-κB activation	(Guelen et al., 2004)
		Hsp70	Inhibit the Hsp70 expression and reduce its transcription	(Chen et al., 2011; Yuan et al., 2013)
		Hsc70	Affects ORF3-induced Akt phosphorylation	(Chen et al., 2011)
PCV	Cytoplasm	pPirh2	Leads to the accumulation of p53 and induction of a caspase cascade to apoptosis in the intrinsic pathway of apoptosis	(Leng et al., 2003; Liu et al., 2007)
		RGS16	Causes ubiquitin-mediated proteasomal degradation of RGS16 and increases NF-κB translocation into the nucleus through the ERK1/2 signaling pathway; induces an increase in IL-6 and IL-8 mRNA transcripts	(Timmusk et al., 2009; Choi et al., 2015)
	/	DDE-like transposase	No more information is available.	(Timmusk et al., 2006)

**Figure 3:**
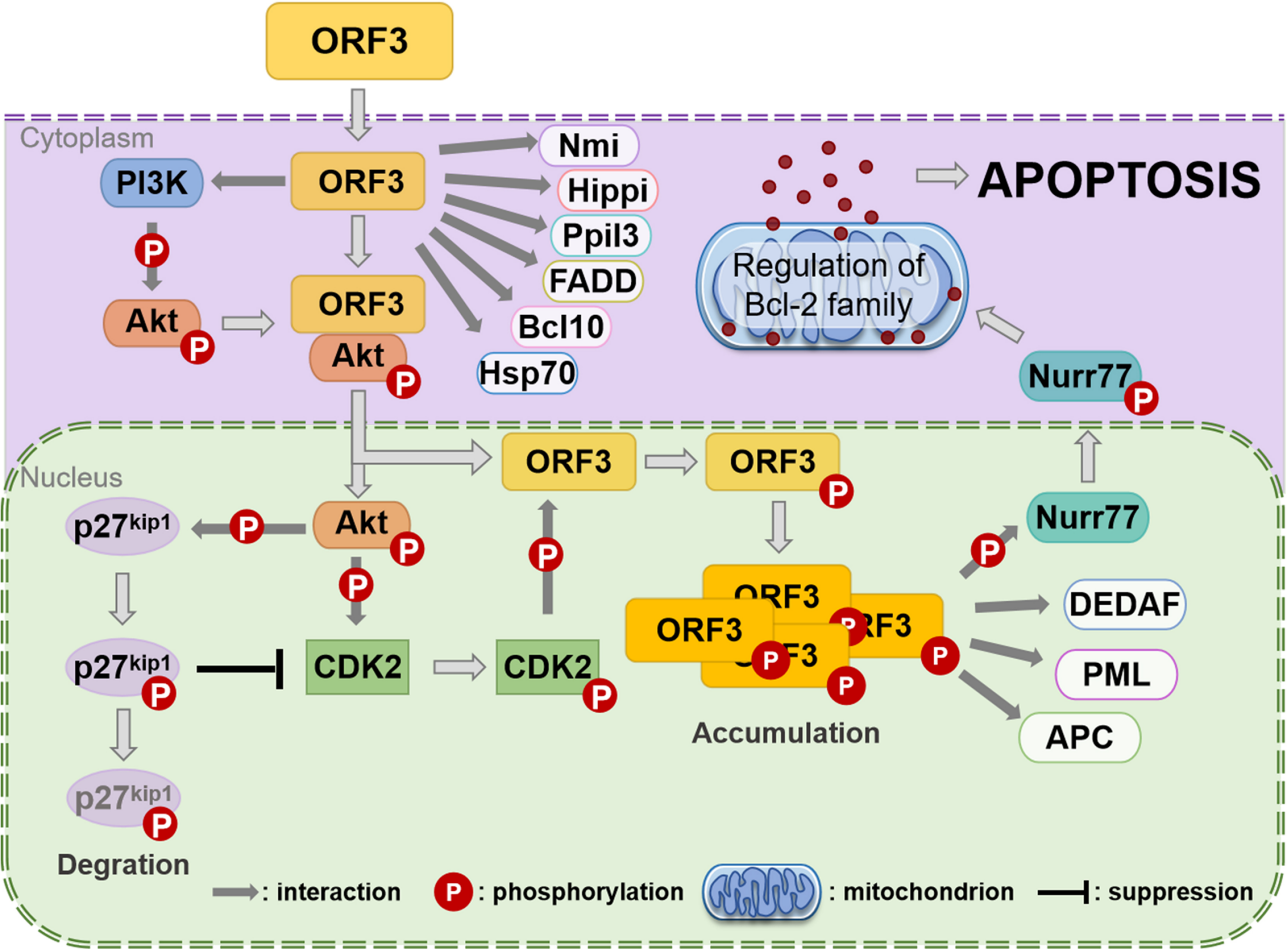
Assumption of the CAV ORF3 protein-induced apoptosis of tumor cells (Los et al., 2009). Through association with PI3-K, the ORF3 protein can lead to constitutive activation of PI3-K and the subsequent phosphorylation of Akt. The nuclear translocation of Akt activates CDK2 by direct and indirect phosphorylation, which subsequently phosphorylates the ORF3 protein at Thr108. Then, the ORF3 protein is forced to accumulate in the nucleus. In the cytoplasm, the ORF3 protein can interact with various proteins, including Nmi, Hippi, Ppil3, FADD, Bcl10, and Hsp70. In the nucleus, the ORF3 protein associates with other types of interaction partners, such as DEDAF, PML and APC. In addition, the ORF3 protein can trigger Nurr77 phosphorylation and then make its nuclear export. Nurr77 in the cytoplasm is known to regulate the Bcl-2 family such that apoptosis is induced *via* the mitochondrial pathway.

It was identified that CAV ORF3 protein interacts with the p85 Src homology 3 (SH3) domain of phosphatidylinositol 3-kinase (PI3-K) in tumor cells (Maddika et al., 2007). The initiation of the PI3-K/Akt pathway triggers the nuclear translocation and activation of Akt, which results in the induction of cyclin-dependent kinase 2 (CDK2), which in turn leads to the phosphorylation of the ORF3 protein (Maddika et al., 2008; Maddika et al., 2009) (**Figure 3**). PKCβ in tumor cells has also been proven to play a crucial role in the phosphorylation and nuclear migration of the CAV ORF3 protein, which induces the activation of multiple signaling events involving caspase 9, caspase 3 activation, and cleavage of PKCδ (Jiang et al., 2010). APC1 is a subunit of the anaphase-promoting complex/cyclosome (APC/C) (Teodoro et al., 2004), which is a major regulator of cell cycle function. Upon ORF3 protein shuttling into the nucleus, APC1 is inhibited, resulting in APC/C disruption, and then, the cell undergoes apoptosis following arrest in the G2/M phase of the cell cycle (Teodoro et al., 2004).

A report provides evidence that the association of ORF3 protein with Ppil3 or Hippi may lead to ORF3 protein sequestration in the cytoplasm and prevent the apoptosis of normal cells (Los et al., 2009). Hippi is a protein that interacts with huntingtin-interacting protein 1 (Hip1) (Cheng et al., 2003), and the Hip-1-Hippi complex has been shown to induce apoptosis through the recruitment and activation of the cysteine protease caspase 8 (Gervais et al., 2002). It has also been shown that both *in vitro* and in human cells, Hippi can bind with the self-multimerization domain of the ORF3 protein, and the ORF3 protein binds to the C-terminal half of Hippi, including its death effector domain-like motif (Cheng et al., 2003). Moreover, in tumor cells, the ORF3 protein and Hippi are primarily located separately in the nucleus and cytoplasm, respectively, whereas in normal cells, they can co-localize in the cytoplasm (Cheng et al., 2003). These features support the theory that, in normal cells, the co-expression of the ORF3 protein and Hippi may suppress the apoptotic activity of the ORF3 protein (Majumder et al., 2006).

#### PCV

**Figure 4:**
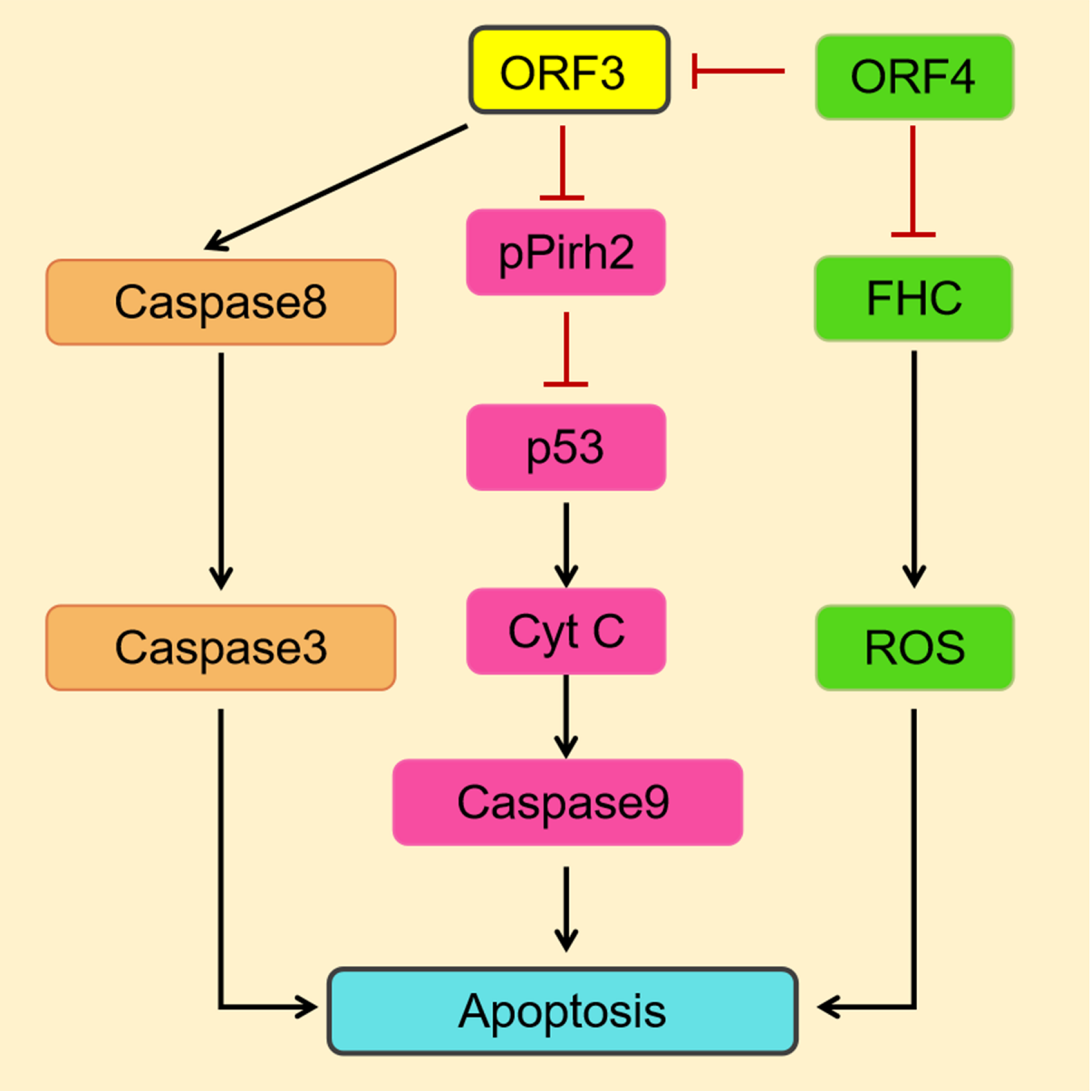
A simulation model describes the mechanisms of PCV2 ORF3-induced apoptosis (Pan et al., 2018). On the one hand, the PCV2 ORF3 protein induces apoptosis through the death receptor pathway *via* the activation of caspase 8 and caspase 3. On the other hand, the interaction of the ORF3 protein and pPirh2 causes the accumulation of p53 and then upregulates factors downstream of p53 to induce apoptosis. The ORF4 protein inhibits apoptosis by suppressing the activation of ORF3. And by interacting with FHC, which has been suggested to prevent cells from undergoing apoptosis induced by a variety of stimuli, it can ultimately suppress apoptosis by inhibiting the production of ROS.

In a study on the modulation of cellular functions by the PCV2, the ORF3 protein was found to interact directly with the E3 ubiquitin ligase pPirh2 (also known as RCHY1) (Liu et al., 2007), which targets p53, a tumor suppressor and a transcription factor (Hong et al., 2014) (**Table 1**). The interaction suppresses pPirh2 stabilization and causes a decrease in degradation of p53, leading to increased accumulation of p53, which eventually induces a caspase signaling cascade that leads to apoptosis *via* the intrinsic pathway (Leng et al., 2003) (**Figure 4**). It was proven that the amino acid residues 20–65 in the ORF3 protein play a crucial role in the interaction of the ORF3 protein with pPirh2, which is competitive over p53 (Karuppannan et al., 2010).

Previously, specific interactions of PCV2 ORF3 with regulator of G protein signaling 16 (RGS16) and DDE-like transposase, the sequence of which is very similar to that of Tn10 transposase sequence, have been reported (Timmusk et al., 2006; Timmusk et al., 2009) (**Table 1**). RGSs, a protein family that modifies signaling *via* G protein-coupled receptors (GPCRs), have a conserved domain of approximately 120 amino acids, which binds to the activated Gα subunit of G-proteins and thereby terminates G-protein signaling (Jean-Baptiste et al., 2006; Xie and Palmer, 2007). More evidence suggests that the direct interaction between PCV2 ORF3 and RGS16 within the cytoplasm leads to the ubiquitin-mediated proteasomal degradation of RGS16, further enhancing NF-κB translocation into the nucleus through the ERK1/2 signaling pathway and increasing the number of IL-6 and IL-8 mRNA transcripts (Choi et al., 2015). However, there is no additional information about the interaction of the DDE-like transposase ORF3 protein.

Currently, studies have indicated that the number of proteins associated with pPirh2 and p53 is greater than 20 and 320, respectively (Jung et al., 2012), which means that the process of ORF3-induced apoptosis may be much more complicated (Pan et al., 2018). Accordingly, future studies may consider whether other factors are involved in regulating apoptosis through their interactions with ORF3 and should determine exactly the roles that the proteins play.

### Pathways of ORF3-Mediated Apoptosis

The precise mechanisms of cell death mediated by the ORF3 protein from the *Circoviridae* family are not clear, although there is a consensus regarding some of the specific molecular events (**Table 1**).

In contrast to PCV and DuCV, the CAV ORF3 protein has the unique ability to induce tumor-specific cell apoptosis independent of p53 (Zhuang et al., 1995). It is now well established that ORF3 protein expression in CAV results in the activation of caspases (Danen-van Oorschot et al., 2000). Moreover, the mitochondrial pathway is activated by the ORF3 protein through a Nurr77-dependent pathway (Maddika et al., 2005) (**Figure 3**). What’s particularly interesting is that Nur77, which is shuttled from the nucleus to the cytoplasm upon the transient expression of ORF3 protein, is able to transmit apoptotic signals from the nucleus to mitochondria (Maddika et al., 2005). Upon transfer to the cytoplasm, Nur77 may directly or indirectly cause cytochrome C and AIF release and activate the mitochondrial apoptosis pathway (Los et al., 2009). Moreover, the data indicates that Apaf-1 is required for ORF3-mediated apoptosis (Burek et al., 2006). Taken together, these studies suggest that the ORF3 protein ultimately induces apoptosis modulated by Bcl2 family members through the loss of mitochondrial membrane potential and the cleavage of caspase 3 and caspase 7 (Maddika et al., 2005; Chaabane et al., 2014).

As opposed to the G2/M arrest observed with the CAV ORF3 protein, PCV1 ORF3 induces dramatic G1 cell cycle arrest (Hough et al., 2015). In contrast to that of PCV2, the ORF3 protein of PCV1 appears to be more cytotoxic by activating a caspase-dependent apoptotic pathway, and potentially initiates a caspase-independent poly ADP-ribose polymerase (PARP) cleavage pathway (Chaiyakul et al., 2010). But the precise apoptotic signaling networks have yet to be discovered. Reports have indicated that the PCV2 ORF3 protein induces apoptosis *via* the death receptor pathway, ultimately by activating both caspase 3 and caspase 8 (Liu et al., 2005) (**Figure 4**), and the apoptotic response is correlated with its nuclear localization (Kiupel et al., 2005). However, in melanoma cells and mouse primary splenocytes, it showed to induce apoptosis in a completely different manner—a p53-dependent pathway, which is independent of caspase 3 and caspase 8 (Teras et al., 2018).

To date, studies have successfully expressed the ORF3 protein of DuCV in DF-1, CHO, and Sf9 cells (Xiang et al., 2012; Wu et al., 2018), which suggests a cellular tendency of ORF3 by performing as a different expression in cell lines. The expression levels of caspase 3 and caspase 8 mRNA are up-regulated after the transfection of ORF3 protein, indicating that the DuCV ORF3 protein may induce apoptosis through the death receptor pathway, similar to PCV2 ORF3. However, the specific mechanism of the signaling pathway remains to be further determined.

## Apoptotic Effects of the Interactions Between ORF3 and Other Viral Proteins

The co-localization and association of the CAV ORF3 and ORF2 protens have been observed. It was revealed that the ORF2 protein directly interacts with the ORF3 protein in the nucleus to downregulate apoptosis by altering the phosphorylation status of the latter, but not completely abolish it (Lai et al., 2017). In the meantime, the de-phosphorylation of ORF3 protein at Thr108 *via* this interaction seems to participate in modulating the CAV infection process (Lai et al., 2017). There is doubt about whether other phosphorylation sites on the ORF3 protein are similarly regulated by ORF2 or if there are more undiscovered sites.

For PCV2, a newly identified putative protein ORF4 was shown to inhibit apoptosis by suppressing the activation of ORF3, as indicated by significant decreases in caspase 3 and caspase 8 (Gao et al., 2014) (**Figure 4**). It was confirmed that the ORF4 protein interacts with ferritin heavy chain(FHC), the only one subunit of ferritin that has ferroxidase activity, resulting in a reduction in FHC content, and ultimately suppresses apoptosis by inhibiting the production of ROS (Lv et al., 2015). On the other hand, the ORF4 protein can induce apoptosis *via* the mitochondrial pathway by interacting with adenine nucleotide translocase 3 (ANT3) (Lin et al., 2018). As stated above, the ORF4 protein has a dual function in the induction of apoptosis, one of which is its association with ORF3 to suppress apoptosis.

## Conclusions and Perspectives

The main histological changes associated with infections by *Circoviridae* family viruses are lymphoid tissues, which show with lymphocyte depletion and necrosis (Woods and Latimer, 2000; Palinski et al., 2016; Dennis et al., 2018; Klaumann et al., 2018) that may be connected to the apoptotic activity of ORF3 proteins. The more studies on ORF3 proteins that are executed, the more evidence confirming its significant role in the pathogenicity of viruses is revealed. Regarding molecular structures, the findings of NLSs in CAV (Rohn et al., 2002), PCV (Lin et al., 2011; Hough et al., 2015), and DuCV (Wu et al., 2018) ORF3 proteins suggest the possibility of these proteins being located in the nucleus. The characterization of the NLSs and NESs of the ORF3 proteins (Wang et al., 2004; Poon et al., 2005; Hough et al., 2015; Gu et al., 2016) has begun to shed light on the subcellular localization of ORF3 proteins, which contributes to their different apoptotic activities. Particularly for the CAV ORF3 protein, phosphorylation sites (Rohn et al., 2002; Rohn et al., 2005; Lanz et al., 2012; Kucharski et al., 2016; Feng et al., 2020), such as Thr108, Thr107, Thr56, and Thr61, are considered to be of vital importance to its nuclear localization and apoptotic effect, whereas it has not yet been discovered in the ORF3 proteins of PCV and DuCV. On the other hand, the CAV ORF3 protein, in particular, has a tumor cell-specific nuclear targeting signal (Danen-Van Oorschot et al., 2003; Kuusisto et al., 2008), which indicates tumor-selective toxicity induced upon its accumulation in the nuclei of transformed cells (Danen-Van Oorschot et al., 1997; Poon et al., 2005; Helck et al., 2008; Zhao et al., 2013). However, whether the ORF3 protein of PCV or DuCV has tumor-selective cytotoxicity remains to be discovered. Hence, these findings suggest that more attention is needed on the aspects highlighted herein to elucidate the mechanisms of the ORF3 proteins.

For the aspect of inducing apoptosis pathways, the PCV2 ORF3 protein activates both caspase 3 and caspase 8 through the death receptor pathway (Liu et al., 2005), which may also be triggered by the DuCV ORF3. The p53-dependent pathway, which is independent of caspase 3 and caspase 8, is considered to be another different manner activated by the PCV2 ORF3 protein. The CAV ORF3 protein is distinct in its ability to induce the selective killing of transformed cells independent of p53 (Zhuang et al., 1995; Noteborn, 2004). More than one-half of human cancers are not responsive to many chemotherapeutics owing to mutations in p53 (Leroy et al., 2014). Therefore, the study on the CAV ORF3 protein provides a unique system for identifying apoptosis pathways (Maddika et al., 2006) that can kill cancer cells selectively independent of p53. Currently, many studies on the CAV ORF3 protein aim to deliver the protein as a potentially safe cancer chemotherapy drug (Ruiz-Martínez et al., 2017; Castro et al., 2018; Wyatt et al., 2019). Clearly, continued studies of pathways related to cell death have obvious therapeutic value (Hough et al., 2015), although the induction activities of the PCV and DuCV ORF3 proteins are weaker than the activity of the CAV ORF3 protein.

In conclusion, although the specific mechanisms of these ORF3 proteins differ in CAV, PCV, and DuCV of the *Circoviridae* family, they are certainly accompanied by the induction of apoptosis (Guelen et al., 2004; Liu et al., 2005; Xiang et al., 2012), which may provide a theoretical foundation to explain their pathogenesis. Based on a wide array of reported works on the CAV, PCV and DuCV ORF3 proteins, this review offers the first glimpse into the ORF3-induced apoptosis by *Circoviridae* viruses, which involves specific structures, subcellular localizations and apoptosis pathways of the ORF3 protein, and suggests that further investigations into these apoptosis mechanisms be researched. As stated above, further study will help us obtain a deeper understanding of the molecular mechanisms of ORF3-induced apoptosis and provide a new perspective on the pathogenesis of viruses in the *Circoviridae* family.

## Author Contributions

YZ conceived and wrote the paper. XZ conceived and modified the paper. RJ, ZY, and JH contributed to English proofreading. MW and AC were responsible for revising the manuscript critically for expert content. All authors contributed to the article and approved the submitted version.

## Funding

This work was supported by the Sichuan Veterinary Medicine and Drug Innovation Group of China Agricultural Research System (CARS-SVDIP), and China Agricultural Research System (CARS-42-17).

## Conflict of Interest

The authors declare that the research was conducted in the absence of any commercial or financial relationships that could be construed as a potential conflict of interest.
